# Safety and Effectiveness of High-Precision Hyperthermic Intraperitoneal Perfusion Chemotherapy in Peritoneal Carcinomatosis: A Real-World Study

**DOI:** 10.3389/fonc.2021.674915

**Published:** 2021-08-06

**Authors:** Mingchen Ba, Shuzhong Cui, Hui Long, Yuanfeng Gong, Yinbing Wu, Kunpeng Lin, Yinuo Tu, Bohuo Zhang, Wanbo Wu

**Affiliations:** ^1^Intracelom Hyperthermic Perfusion Therapy Center, Affiliated Cancer Hospital & Institute of Guangzhou Medical University, Guangzhou, China; ^2^Department of Pharmacy, Guangzhou Dermatology Institute, Guangzhou, China

**Keywords:** high-precision, hyperthermic intraperitoneal perfusion chemotherapy, peritoneal carcinomatosis, design, a real-world study

## Abstract

**Background:**

Hyperthermic intraperitoneal chemotherapy (HIPEC) has been reported to effectively control peritoneal carcinomatosis (PC) in various patient populations, but there is a lack of real-world data. This study aimed to examine the safety and effectiveness of HIPEC in patients with PC in a real-world setting.

**Methods:**

This was a retrospective study of patients with PC treated with the high-precision BR-TRG-I type HIPEC device between December 2006 and December 2016. Vital signs during HIPEC and adverse events were recorded. Effectiveness was evaluated by total objective remission rate (ORR), which was based on ascites’ remission 4 weeks after HIPEC.

**Results:**

A total of 1,200 patients were included. There were 518 males and 682 females, with a mean age of 58.6 ± 6.5 years (range, 32–76 years). Among the patients, 93.6% of the patients (1123/1200) successfully received the three sessions of HIPEC, 158 had massive ascites. The changes of vital signs during HIPEC were within acceptable ranges, and patients only had a transient fever and abdominal distension. Regarding the HIPEC-related complications, hemorrhage was observed in seven (0.6%) patients, anastomotic leakage in four (0.5%), and intestinal obstruction in eight (0.7%). Nine (0.8%, 9/1200) patients had CTCAE grade IV bone marrow suppression, and three (0.3%, 3/1200) patients had severe renal failure (SRF), which were considered to be drug-related. The ORR of malignant ascites was 95.6% (151/158).

**Conclusion:**

This real-world study strongly suggests that HIPEC was safe in treating PC patients with a low rate of adverse events and leads to benefits in PC patients with massive malignant ascites.

## Background

Peritoneal carcinomatosis (PC) is a regional disseminated invasion of the peritoneal cavity by cancer cells of various origins, including colorectal cancer, gastric cancer, and epithelial ovarian cancer (1). PC affects overall survival (OS) unfavorably, even in the absence of obvious metastases at other sites (1–3). PC is often considered a terminal condition because systemic chemotherapy has a marginal effect on PC (4, 5). Nevertheless, removing the peritoneal seeds has been shown to improve prognosis (1, 6), but surgery alone cannot be conducted at the microscopic level (4, 5).

Hyperthermic intraperitoneal chemotherapy (HIPEC) has been reported to have satisfactory effectiveness in treating PC and malignant ascites secondary to PC (7–10). Because PC represents a disseminated regional disease without proof of distant metastases (11), it can be treated with HIPEC regional therapy to prolong patients’ survival and improve prognosis (11). In HIPEC, the chemotherapeutic agents circulate into the abdominal cavity with a high, constant, and persistent drug concentration, with only small amounts of drugs entering the blood circulation system, which take full advantage of the synergism of hyperthermia and cytotoxic drugs and the pharmacokinetic advantage of intraperitoneal chemotherapy, and have fewer adverse effects than systemic chemotherapy (12–14). Thus, as a new adjunctive therapy against PC, HIPEC has obvious advantages compared with simple systemic chemotherapy in terms of prevention and treatment of peritoneal metastasis from malignant tumors (15–17).

The main problem with HIPEC is that many kinds of devices used all over the world have issues, such as poor temperature control precision, unstable perfusion rate, and complicated application. As a result, the HIPEC applicability, usefulness, and feasibility of HIPEC failed to reach clinical standards, and HIPEC is associated with a relatively high complication rate (18–24). These previous conventional devices were based on hot water-, microwave-, or radiofrequency-induced local hyperthermia, and have uncertain flow control accuracy, uncertain temperature accuracy, and 1°C to 2°C temperature control accuracy. Thus, there are large discrepancies in reported HIPEC-related complications morbidity (0–39%) and mortality rates (0–20%), regardless of indication, technique, and cytotoxic drugs used (25–31). To overcome the previous HIPEC devices’ issues, we developed a high-precision HIPEC device: the “BR-TRG-I-type hyperthermic intraperitoneal perfusion chemotherapy device” (BR-TRG-II; Guangzhou Baorui Medical Instrument Co., Ltd., Guangzhou, China). This novel device has been designed to achieve a high-precision regarding temperature and flow rate.

This device has been shown to effectively control PC in various patient populations (22, 23, 32, 33), and there is a lack of large sample-sized study in a clinical-practice setting. Therefore, the present real-world study aimed to examine the safety and effectiveness of HIPEC in patients with PC. The results could provide a novel method to treat PC.

## Patients and Methods

### Study Design and Patients

This was a retrospective real-world study of PC patients originating from gastric cancer, colorectal cancer, ovarian cancer, or pseudomyxoma peritonei and treated with HIPEC using the high-precision BR-TRG-I type HIPEC device between December 2006 and December 2016. The patients were from four hospitals in China: Affiliated Cancer Hospital & Institute of Guangzhou Medical University, Affiliated Cancer Hospital of Sun Yat-Sen University, The Second Affiliated Hospital of Guangzhou Medical University, and The First Affiliated Hospital of Sun Yat-Sen University. The study was approved by the Ethics Committees of the four participating hospitals, with the Affiliated Cancer Hospital & Institute of Guangzhou Medical University as the lead center (approval GZMCY20080825). The requirement for written informed consent was waived by all four committees because of the retrospective nature of the study.

The inclusion criteria were 1) ≥18 years of age, 2) no radiation therapy in the previous 4 weeks, 3) no systemic chemotherapy or intraperitoneal chemotherapy in the previous 2 weeks, and 4) the tumor board determined that the prognosis was >2 months. The exclusion criteria were 1) known or possible metastasis from tumors in other organs, 2) recurrent colorectal cancer with ascites, 3) known or possible malignant tumor in other internal organs, 4) active inflammation or infection, 5) extensive intraperitoneal adhesions due to multiple operations, 6) malignant ascites with complete obstruction, or 7) cachexia.

### Device Design

**Figure 1:**
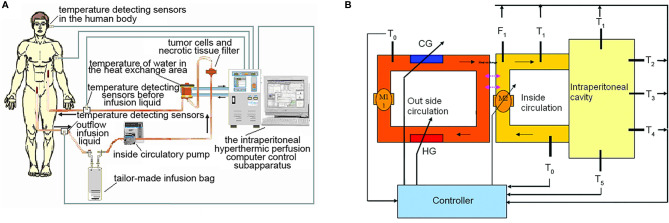
The BR-TRG-I-type intraperitoneal hyperthermic perfusion chemotherapy device. Sketch map and stucture chart. **(A)** Sketch map, **(B)** stucture chart. HG: heater, with a maximum power of 4 kW; adjustable heating current. CG: semi-conductor refrigerator, with a maximum power of 2 kW; adjustable refrigerating power. T°: temperature of water in the external heat exchanger, with a precision of 0.1°C. Tii: temperature of the internal circulatory perfusate before entering the body, precision of 0.1°C. Ti°: temperature of the internal outflow perfusate, with a precision of 0.1°C. Fi: flow rate of the internal circulatory perfusion liquid, with a precision of 10 ml/min; resolution of 1 ml/min. P: the pressure of the internal perfusate before entering the human body, with a precision of 10 mmHg; resolution of 1 mmHg. T_1_-T_5_: temperature of five important parts of the human body. M1: external circulatory pump, constant working rate of 10 L/min. M2: internal circulatory pump, rotating rate, maximum controllable rate, 600 ml/min; precision of 10 ml/min; resolution of 1 ml/min.

**Figure 2:**
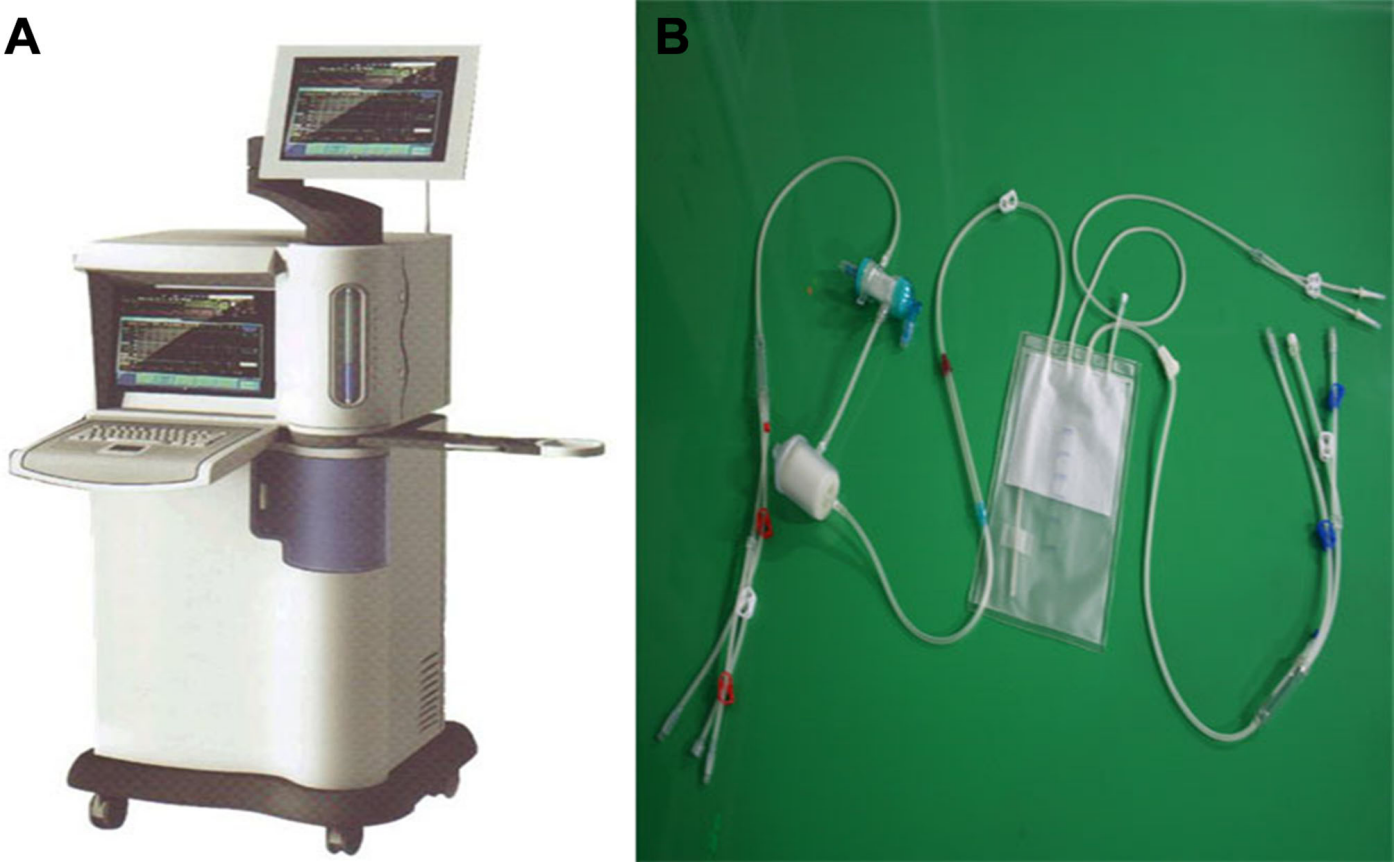
BR-TRG-I type high-precision hyperthermic intraperitoneal perfusion chemotherapy (HIPEC) treatment system by way of BHPC application. **(A)** The BR-TRG-I type high precision hyperthermic perfusion intraperitoneal treatment device. **(B)** The BR-TRG-II type high precision hyperthermic perfusion intraperitoneal treatment pipeline.

The high precision “BR-TRG-I-type hyperthermic intraperitoneal perfusion chemotherapy device” was designed as an ambulatory device that can be easily moved within the hospital, if needed. It was designed with ±0.10°C temperature measurement accuracy, ± 0.10°C temperature control accuracy, and ±5% flow control precision, and an automatic temperature control system, including both automatic heating and cooling functions. The device can also automatically record all treatment-related data, processing data, and performing an automatic cooling function. The preheating, heating, temperature control, and cooling performances of the device met the above design requirements. **Figure 1** shows a schematic of the device. The high-precision BR-TRG-I-type HIPEC device is protected by two patents from China: Intraperitoneal Hyperthermic Perfusion Treatment Apparatus (patent number ZL2006200613779) and Continuous Intraperitoneal Hyperthermic Perfusion Treatment Apparatus (patent number ZL2006200613764). The device has been approved by the SFDA of China (number 2009-3260924) and is the only approved class III intraperitoneal hyperthermic perfusion device in China (22–26) (**Figure 2**).

### HIPEC

**Figure 3:**
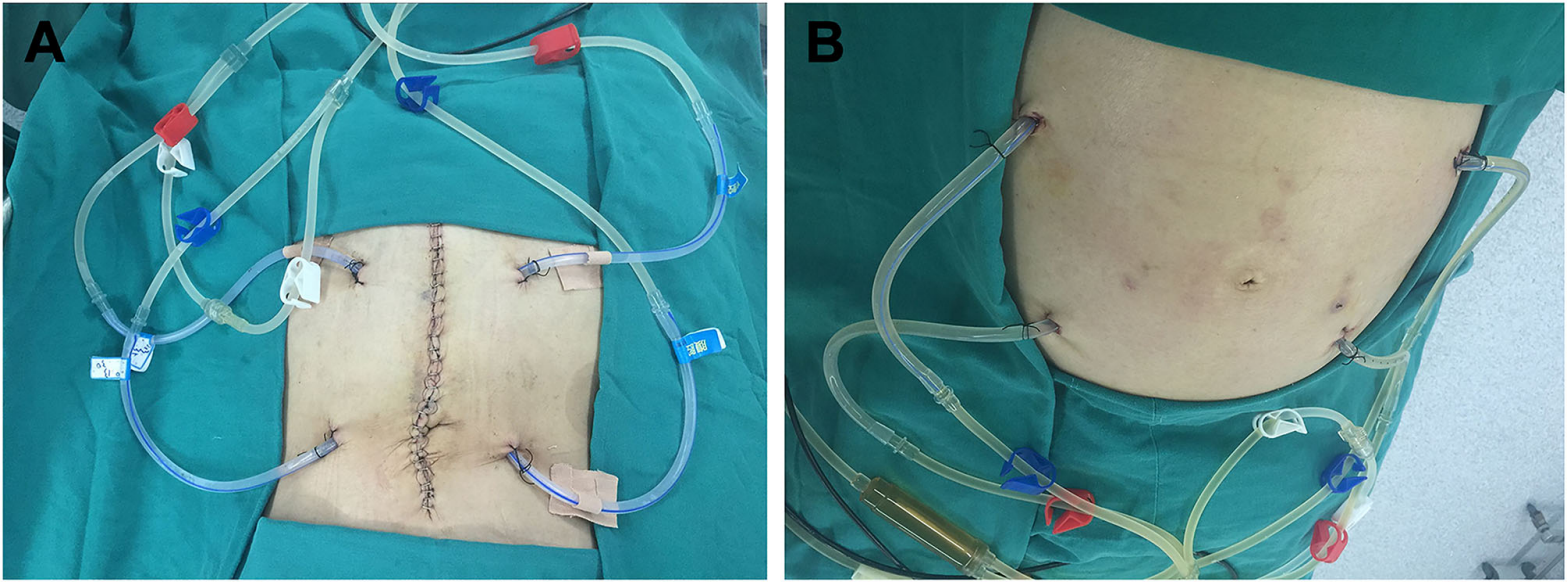
Placement sites of the infusion and outflow catheters for HIPEC followed by dCRS. **(A)** Localization of the infusion and outflow catheters for HIPEC followed by dCRS. **(B)** Localization of the infusion and outflow catheters for HIPEC followed by minimally invasive surgery. The red clips mark the two infusion catheters. The blue clips mark the two outflow catheters. The white clips mark the loop circuit for HIPEC preparation.

All patients were attempted to receive three sessions of perioperative HIPEC after cytoreductive surgery or placement of the perfusion tubing by minimally invasive surgery. The first session of HIPEC was completed in the operating room under anesthesia immediately after placement of the infusion tubes. The infusion and outflow catheters were placed, as shown in **Figure 3**. The second and third sessions were performed in the intensive care unit (ICU) or the operating room on the first and second days after the first session of HIPEC. Pethidine hydrochloride (75 mg) and promethazine hydrochloride (25 mg) were administered by intramuscular injection before HIPEC. Propofol (3–12 mL/h) was intravenously injected as an anesthetic agent with a continuous vein pump for sedative anesthesia. The dose was continuously adjusted according to patient status.

The chemotherapeutic agent and their dosage were selected according to the primary tumor and body weight. Oxaliplatin (125 mg/m^2^, Jiangsu Hengrui Medicine Co., Ltd., Nanjing City, Jiangsu Province, China) and carboplatin (350 mg/m^2^, Yangzi River Pharmaceutical Co. Ltd., Nanjing City, Jiangsu Province, China) were diluted in 5% glucose (3,000–7,000 ml). Paclitaxel (75 mg/m^2^; Jiangsu Aosaikang Pharmaceutical Co Ltd, Nanjing City, Jiangsu Province, China) with 5-fluorouracil (1500 mg/m^2^, Yangzi River Pharmaceutical Co., Ltd., Nanjing City, Jiangsu Province, China), or cisplatin (80 mg/m^2^; Jiangsu Haosen Pharmaceutical Ltd. Co., Nanjing City, Jiangsu Province, China) with 5-fluorouracil (1500 mg/m^2^, Yangzi River Pharmaceutical Co., Ltd., Nanjing City, Jiangsu Province, China) were diluted in 0.9% saline (3,000–7,000 ml). The perfusion duration was 90 min. The inflow temperature was set as 43°C.

The BR-TRG-II treatment system was preheated to 40°C to begin treatment as the starting point for treatment (0 min). The treatment temperature during HIPEC was monitored by the BR-TRG-II treatment system using temperature-monitoring probes in the inflow and outflow catheters. The patient’s vital signs were assessed (G3HJ20025, Shenzhen Medical Instrument Co., Ltd., Shenzhen, China). After the third HIPEC session, all ascites were drained out, and the infusion catheters were removed. The outflow catheters were kept for 3 to 5 days as drainage catheters.

### Outcomes and Follow-Up

Any adverse events and reasons for stopping treatment were recorded. The toxic effects of anticancer drugs were graded according to the National Cancer Institute Common Toxicity Criteria for Adverse Events (CTCAE) Version4.0. Effectiveness was evaluated based on ascites remission according to our previous modification of the World Health Organization criteria (22–26). Calculation of the total objective remission rate (ORR) was based on the remission of ascites 4 weeks after the completion of HIPEC (23). A complete response (CR) was defined as the absorption of the ascites at 4 weeks after treatment. A partial response (PR) was defined as the absorption of 50% of the ascites at 4 weeks after treatment. No consequence (NC) was defined as no obvious reduction after treatment. The ORR was CR+PR. The data of overall survival were collected as well.

### Statistical Analysis

Data were analyzed using SPSS 17.0 (SPSS Inc., Chicago, USA). All continuous data are presented as mean ± standard deviation (SD) or median (range) and were analyzed using analysis of variance (ANOVA) and the Student’s t-test, with Bonferroni correction for multiple comparisons to compare the results before and after therapy. Categorical data are presented as n (%) and were analyzed using the chi-square test. Two-sided P-values <0.05 were considered statistically significant.

## Results

### Characteristics of the Patients

**Figure 4:**
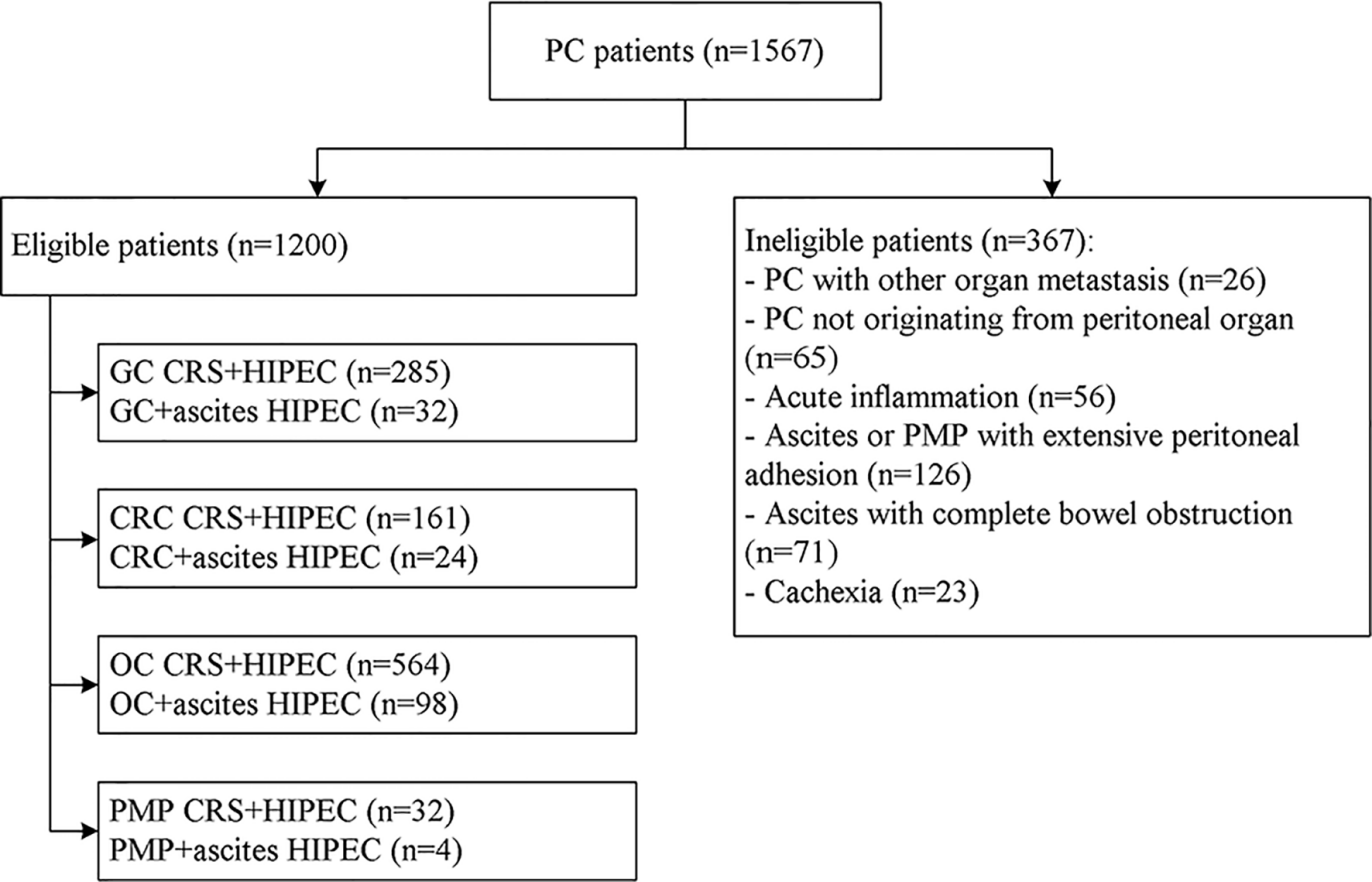
Flowchart of the patient recruitment for preliminary clinical application of the high-precision hyperthermic intraperitoneal perfusion chemotherapy device. GC, gastric cancer; CRC, colorectal cancer; OC, ovarian cancer; PMP, pseudomyxoma peritonei; CRS, cytoreductive surgery.

The process of inclusion and exclusion were presented in **Figure 4**. Finally, a total of 1200 PC patients with or without malignant ascites were included. There were 518 males and 682 females, with a mean age of 58.6 ± 6.5 years (range, 32–76 years). The most common conditions were ovarian cancer (n=564, 47.0%) and ovarian cancer with massive ascites post cytoreductive surgery (n=98, 8.2%), gastric cancer with PC (n=285, 23.75%), gastric cancer with massive ascites post cytoreductive surgery (n=32, 2.7%), colorectal cancer (n=161, 47.0%), and colorectal cancer with massive ascites post cytoreductive surgery (n=24, 8.2%). Among the 1200 patients, 158 (13.2%) had massive ascites, and the volume of ascites was 4624 ± 231 ml. The disease course from disease diagnosis to admission was 15.5 ± 6.6 days, and 618 cases (51.5%) could find malignant cells in the abdominal cavity (**Table 1**).

**Table 1:** Characteristics of the patients.

Characteristics	Total (n=1200)
Age, years, mean ± SD	58.6 ± 6.5
Male, n (%)	518 (43.2)
Diagnosis and surgery before HIPEC, n (%)	
Ovarian cancer	564 (47.0)
Ovarian cancer post cytoreductive surgery with massive ascites	98 (8.2)
Gastric cancer	285 (23.8)
Gastric cancer post cytoreductive surgery with massive ascites	32 (2.7)
Colorectal cancer	161 (13.4)
Colorectal cancer post cytoreductive surgery with massive ascites	24 (2.0)
Pseudomyxomaperitonei	25 (2.4)
Pseudomyxomaperitonei with massive ascites	4 (0.3)
Pancreatic cancer with massive ascites	7 (0.6)
Disease course, days, mean ± SD	15.5 ± 6.6
Ascites volume, ml, mean ± SD	4624 ± 231
Patients with massive ascites, n (%)	158 (13.2)
Cases of free cancer cells in the abdominal cavity, n (%)	618 (51.5)

HIPEC, hyperthermic intraperitoneal chemotherapy; SD, standard deviation.

Among the included patients, 93.6% of the patients (1123/1200) successfully received the three sessions of HIPEC. The remaining 77 patients could not receive the three sessions of HIPEC because of outflow catheter obstruction (n=28), the perfusion fluid did not flow from the peritoneal cavity or flowed slowly (n=35), patients could not tolerate abdominal distension due to perfusion liquid accumulation (n=12), and automatic emergency safety stop of the perfusion system (n=2).

### Vital Signs During HIPEC

**Figure 5:**
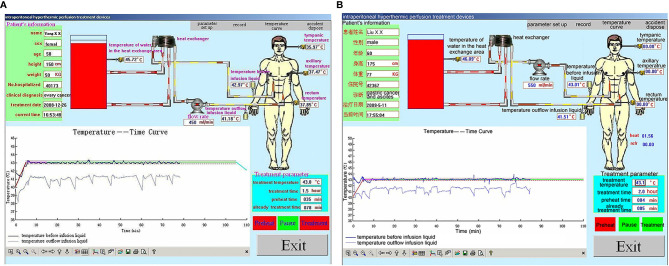
Representative performance graphs of the temperature control of the BR-TRG-I-type intraperitoneal hyperthermic perfusion chemotherapy (HIPEC) device in two patients at the first HIPEC session. **(A)** HIPEC followed by dCRS. **(B)** HIPEC followed by minimally invasive surgery. The graphs show that the temperature in the infusion and outflow catheters was stable at 43°C and 41.8°C. The performance was similar between the two approaches.

During HIPEC treatment, variations in vital signs (including respiratory rate) were observed in many patients from the beginning of HIPEC to the end of HIPEC (90 min). Nevertheless, the patients showed no sign of discomfort, but they had a transient fever and abdominal distension. During HIPEC, the temperature in the inflow and outflow catheters was 43°C and 41.8°C, and the maximum tympanic temperature was <37.5°C for the whole 90-min procedure (P=0.07, P=0.10, and P=0.08, respectively). In all patients, the blood oxygen saturation values were maintained within acceptable standard ranges, and blood pressure changes were mild (**Figure 5**). In all included patients, rectum temperature and heart and respiration rates were higher than the baseline values during HIPEC (90 min) (P=0.001 and P=0.001). Nevertheless, the procedure was tolerated by all patients. All parameters returned to baseline by 30 min after HIPEC (**Table 2**).

**Table 2:** Effect of HIPEC on vital signs using the BR-TRG-I-type intraperitoneal HIPEC.

	Time (min)					P
	0	30	60	90	120	
Perfusion tubes temperature (°C)	40.0 ± 0.2	43.0 ± 0.2	43.0 ± 0.2	43.0 ± 0.2	40.0 ± 0.2	0.07
Outflow tubes temperature (°C)	39.6 ± 0.7	41.9 ± 0.2	42.0 ± 0.2	42.0 ± 0.1	39.7 ± 0.2	0.001
Rectum temperature (°C)	36.8 ± 0.2	39.9 ± 0.2	40.2 ± 0.3	40.0 ± 0.2	36.8 ± 0.2	0.001
Tympanic membrane temperature (°C)	36.3 ± 0.1	37.5 ± 0.3	37.7 ± 0.3	37.7 ± 0.3	37.6 ± 0.3	0.06
Blood oxygen saturation values (°C)	98 ± 2	99 ± 1	99 ± 1	98 ± 2	98 ± 1	0.08
Systolic blood pressure (mmHg)	118 ± 6	119 ± 6	109 ± 8	110 ± 7	109 ± 7	0.05
Diastolic pressure (mmHg)	73 ± 4	81 ± 5	87 ± 3	89 ± 4	78 ± 5	0.02
Heart rate (bpm)	84 ± 3	110 ± 45	108 ± 3	114 ± 6	88 ± 6	0.01
Respiration rate (breaths/min)	17 ± 1	19 ± 1	24 ± 1	23 ± 2	17 ± 2	0.01

### Adverse Events

No postoperative deaths and complications such as intra-abdominal infection or adhesive intestinal obstruction. Regarding the HIPEC-related complications, hemorrhage was observed in seven (0.6%) patients, anastomotic leakage in four (0.5%), and intestinal obstruction in eight (0.7%). There were no deep abdominal abscesses or peritonitis. Regarding the drug-related complications, 13 (1.1%) patients had leukopenia requiring GM-CSF, seven (0.6%) had anemia requiring blood transfusion, and five (0.4%) had thrombocytopenia. Nine (0.8%, 9/1200) patients had CTCAE grade IV bone marrow suppression, and three (0.3%, 3/1200) patients had severe renal failure (SRF). All these cases were ruled to be due to the chemotherapeutic agents that probably found a way to enter the systemic circulation (**Table 3**).

**Table 3:** HIPEC- and drug-related adverse events.

Adverse events	Total (n=1200)
HIPEC-related	
Hemorrhage	7 (0.6%)
Anastomotic leakage	4 (0.5%)
Deep abdominal abscess or peritonitis	0
Intestinal obstruction	8 (0.7%)
Drug-related	
Leukopenia requiring GM-CSF treatment	13 (1.1%)
Anemia requiring blood transfusion	7 (0.6%)
Thrombocytopenia	5 (0.4%)
Severe complications	
Grade IV bone marrow suppression	9 (0.8%)
Severe renal failure	3 (0.3%)

Data displayed as n (%).

HIPEC, hyperthermic intraperitoneal chemotherapy; GM-CSF, granulocyte-macrophage colony-stimulating factor.

### Effectiveness

Among the 158 patients who had massive ascites and could be included in the effectiveness evaluation, clinical CR of ascites was achieved in 128 patients (81.01%), PR was achieved in 23 patients (14.55%), and no effectiveness was observed in 7 patients (4.43%). The total objective remission rate of this study was 95.6%.

Except for 2 patients with gastric cancer and massive ascites, and 1 patient with colon cancer and massive ascites were treated with HIPEC again 4, 6, and 6 months respectively after first HIPEC treatment course, no recurrence of ascites occurred in other patients until all death in other patients.

Patients with ovarian cancer and peritoneal carcinomatosis with massive ascites post-ovarian cancer cytoreductive surgery have survival time from 7 to 168 months with a median survived time of 36 months, and two patients with ovarian cancer and peritoneal carcinomatosis had a disease-free survival for more than 10 years; patients with gastric cancer and peritoneal carcinomatosis post-gastric cancer resection have survival time from 2 to 18 months with a median survived time of 6.5 months; patients with colorectal cancer and peritoneal carcinomatosis post colorectal cancer resection have survival time ranged 5 to 21 months with a median survival time of 8.5 months. Furthermore, there was a significant difference in survival time of patients with different types of cancers (p<0.001).

## Discussion

HIPEC for PC has been reported to produce satisfactory effects, but there are wide discrepancies among the tried devices in terms of benefits and adverse effects (25–31). HIPEC has also been reported to control PC in various patient populations effectively, but there is a lack of large sample-sized studies in a clinical-practice setting. This real-world study shows a low rate of adverse events in HIPEC application and benefits in PC patients with massive malignant ascites.

Several reports indicate that HIPEC shows significant clinical benefits for PC treatment (18–21). Nevertheless, HIPEC requires suitable equipment for peritoneal perfusion and temperature control adjustment to achieve reliable and repeatable results. HIPEC could not achieve satisfactory therapeutic results using the previously available systems and has been associated with a relatively high complication rate (22–24, 34, 35). The BR-TRG-I-type HIPEC device developed by our team can adapt to the changes in the patient’s parameters and environment. The temperature control system has a good ability of learning and adaptive control and achieves good control. The test results for the approval by the SFDA of China showed the high precision of the BR-TRG-I-type HIPEC device, which is within the design parameters of ±0.10°C temperature reading, and temperature control precision and ±5% flow control precision, which is much more precise than the other systems used in other centers all over the world. Indeed, these other systems are associated with some uncertainty of 1°C to 2°C of temperature measurement and control accuracy, as well as uncertain flow control accuracy (25–31, 34, 35).

Synergism between various cytotoxic drugs and hyperthermia is the theoretical basis of HIPEC in the prevention and treatment of peritoneal metastases. Still, there is no consensus yet on the optimal treatment temperature during HIPEC. Some studies demonstrated that this synergism between various cytotoxic drugs and hyperthermia is stronger at temperatures >39°C. On the other hand, the rate of small bowel heat injury increases above 43°C in humans, while HIPEC at ≤43°C appears to not influence the complication rate of HIPEC (11, 24). The longer the peritoneal cavity temperature remains near 43°C, the stronger the synergism between cytotoxic drugs and hyperthermia. Hence, the maintenance of the peritoneal cavity temperature near 43°C during HIPEC is the technical core of the HIPEC device, and temperature measurement and control accuracy are the keys of HIPEC to achieve satisfactory clinical effects. Therefore, most groups performed HIPEC at temperatures of 41°C to 43°C for 60 to 90 min or longer (14, 15, 18, 19, 25–31). The BR-TRG-I-type HIPEC device uses a complex neural network adaptive control system based on the fuzzy theory to solve temperature control precision. It is proved that when using the neural network adaptive control system, the output of the neural network controller can adapt to the changes in the patient’s parameters and environment.

On the other hand, less is known about the clinical impact of the flow rate control accuracy. Because the fluid continuously recirculates within the peritoneal cavity for 90 min, we can be sure that the entire peritoneal cavity has been exposed to the full dose of chemotherapeutic drugs. Nevertheless, a too high flow rate could increase the peritoneal pressure and lead to adverse effects. The BR-TRG-I-type HIPEC device is equipped with safety features that alarm the personnel if the pressure increases above 20 mmHg and stop perfusion if the pressure increases above 25 mmHg. Nevertheless, it is known that an intra-abdominal pressure >25 mmHg has harmful effects and is an indication for surgical decompression (36, 37). Because intra-abdominal hypertension is defined as pressure >12 mm Hg (37), and because intra-abdominal pressure varies during treatment and is probably >12 mm Hg, the outflow tubes are retained for 3 days after the last HIPEC treatment to allow for drainage of the perfusion fluid and to prevent intra-abdominal hypertension. Nevertheless, the low rate of systemic complications in our 1200 patients (0.75% for grade IV bone marrow suppression and 0.25% for SRF) suggests the good control rate of perfusion flow and intra-abdominal pressure using this device. Still, we cannot exclude that the possibility of some chemotherapy entering the systemic circulation in these nine patients was, in fact, due to a too high intra-abdominal pressure. Additional studies are necessary on this issue.

Importantly, among the evaluated patients before and after treatment for their ascites, the ORR was 95.4%, as supported by previous studies that suggested a high response rate to HIPEC (18–21). The overall survival was 36 months (range: 7–168 months), 6.5 months (range: 2–18 months), 8.5 months (range: 5–21 months) and 8.5 months (range: 5–21 months) for patients with ovarian cancer, gastric cancer, colorectal cancer and pseudomyxomaperitonei, respectively. Due to the lack of comparison, the effectiveness of HIPEC contributed to the prolonged OS remained unclear. Further studies are needed to confirm the effect of HIPEC on the survival of individual kind of malignancy.

The clinical application showed that most of the patients in this study could successfully receive the three HIPEC sessions. Only a few patients could not complete the treatment because of drainage tube obstruction. No patient had to stop treatment because of device failure. No postoperative deaths and complications such as intra-abdominal infection, adhesive intestinal obstruction, or other complications related to the procedure occurred among the 1,200 patients. As discussed above, there was 1% of grade IV toxicities, but the exact cause is yet to be determined. Moreover, the device had no particular requirement in the working environment. Taken together, these results demonstrated that the device completely satisfied the clinical design requirements.

The present study is not without limitations. First, the proportion of patients with evaluated ascites was small, limiting subgroup analyses and observing the HIPEC pathological changes of peritoneal organs at different time points. Besides, although the OS of all kinds of malignant peritoneal tumors were presented, whether the effectiveness of HIPEC contributed to the prolonged OS remained unclear. Therefore, multicenter studies are necessary to address these issues correctly.

In conclusion, this real-world study strongly suggests that HIPEC was safe in treating PC patients with a low rate of adverse events and leads to benefits in PC patients with massive malignant ascites. This device has good prospects for the management of PC.

## Data Availability Statement

The raw data supporting the conclusions of this article will be made available by the authors, without undue reservation.

## Ethics Statement

The study was approved by the Ethics Committees of Affiliated Cancer Hospital & Institute of Guangzhou Medical University (approval #GZMCY20080825). The patients/participants provided their written informed consent to participate in this study.

## Author Contributions

MB, SC, HL, and YG carried out the studies, participated in collecting data, and drafted the manuscript. YT, BZ, and WW performed the statistical analysis and participated in its design. YW and KL helped to draft the manuscript. All authors contributed to the article and approved the submitted version.

## Funding

This study was supported by grants from the Guangzhou key medical discipline construction project (No. 2017), the Guangdong science and technology plan project (No. 20160918), the Guangzhou Science Technology and Innovation Commission (No. 2014Y2-00152), and the Guangzhou Science Technology and Innovation Commission (No. 2014Y2-00548). The funders had no role in study design, data collection, and analysis, decision to publish, or preparation of the manuscript.

## Conflict of Interest

The authors declare that the research was conducted in the absence of any commercial or financial relationships that could be construed as a potential conflict of interest.

## Publisher’s Note

All claims expressed in this article are solely those of the authors and do not necessarily represent those of their affiliated organizations, or those of the publisher, the editors and the reviewers. Any product that may be evaluated in this article, or claim that may be made by its manufacturer, is not guaranteed or endorsed by the publisher.

## Abbreviations

HIPEC, Hyperthermic intraperitoneal perfusion chemotherapy; ICU, intensive care unit; NCI-CTCAE, National Cancer Institute Common Toxicity Criteria for Adverse Events; ORR, objective response rate; PC, peritoneal carcinomatosis; SD, standard deviation; DRF, severe renal failure.
